# Effects of Greenness on Myopia Risk and School-Level Myopia Prevalence Among High School–Aged Adolescents: Cross-sectional Study

**DOI:** 10.2196/42694

**Published:** 2023-01-09

**Authors:** Chang Zhang, Cheng Wang, Xin Guo, Huiyu Xu, Zihao Qin, Liyuan Tao

**Affiliations:** 1 Research Institute of Forestry Chinese Academy of Forestry Beijing China; 2 Institute of School Health Beijing Center for Disease Prevention and Control Beijing China; 3 School of Public Health Capital Medical University Beijing China; 4 Faculty of Medicine University of Queensland Brisbane Queensland Australia; 5 Research Center of Clinical Epidemiology Peking University Third Hospital Beijing China; 6 Medical Examination Centre Peking University Third Hospital Beijing China

**Keywords:** high school–aged adolescent, personal myopia risk, school-level myopia prevalence, green space, adjusted effects

## Abstract

**Background:**

Myopia is a serious public health issue. High school–aged adolescents in Beijing have an alarming prevalence of myopia. Therefore, determining myopia protective factors is essential. Green space has a certain association with myopia protective factors that can protect against myopia.

**Objective:**

This study aims to examine the effects of green space around schools on individual myopia risk in high school–aged adolescents and the school-level myopia prevalence.

**Methods:**

Green space was measured using the normalized difference vegetation index (NDVI). A total of 13,380 samples of 51 high schools were selected from a 2021 Beijing Municipal Health Commission survey. Adolescent myopia was defined as a spherical equivalent of ≤–1.00 diopters in the worse eye. Generalized linear mixed models with a binomial error structure were used to analyze the effects of the NDVI on personal myopia risk and adjust them by other factors, such as demographics, exposure time, and outdoor exercise. The effects of the NDVI on school-level myopia prevalence with adjusted demographics and the relative position factors of trees were analyzed through quasibinomial regression.

**Results:**

The overall prevalence of myopia was 80.61% (10,785/13,380, 95% CI 79.93%-81.27%). Although with a 0.1 increase in the 500 and 1000 m buffer NDVIs adjusted by demographic and other factors, the high school–aged personal myopia risk significantly dropped by 16% (odds ratio [OR] 0.84, 95% CI 0.73-0.97) and 12% (OR 0.88, 95% CI 0.79-0.99), respectively. However, only the adjusted 500 m buffer NDVI (by demographics and the position of trees) with a 0.1 increase significantly reduced the school-level myopia prevalence by 15% (OR 0.85, 95% CI 0.74-0.98). Subgroup analysis showed that the adjusted effects of the 500 m buffer NDVI are significant in schoolgirls (OR 0.82, 95% CI 0.72-0.93), juniors (OR 0.82, 95% CI 0.72-0.94), the Han nationality (OR 0.84, 95% CI 0.72-0.97), 1-year exposure (OR 0.84, 95% CI 0.71-0.99) and 3-year exposure (OR 0.78, 95% CI 0.65-0.94).

**Conclusions:**

The greenness of a 500 m buffer around schools is associated with a lower personal myopia risk among adolescents and a lower prevalence of myopia in schools. With regard to prevention and control activities, green space within a 500 m buffer around schools is suggested as an independent protective factor for adolescent myopia.

## Introduction

**Figure 1:**
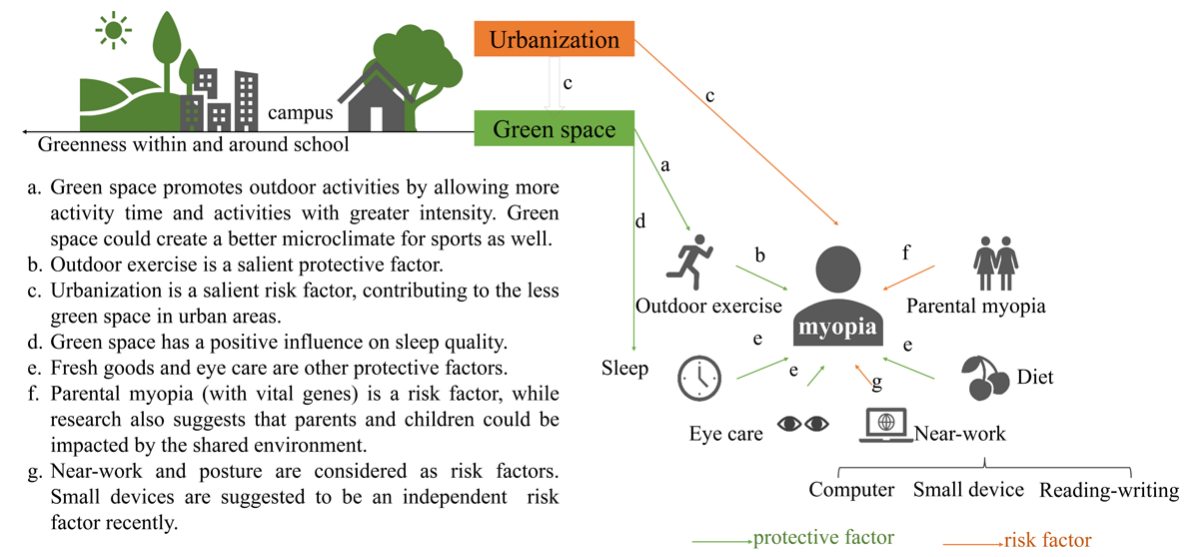
Based on previous studies, greenness may play a role in vision protection against myopia. The green and orange arrow lines indicate protective and risk factors according to previous studies, respectively. Points b, c, e, f, and g represent the influencing factors on myopia, while points a and d explain the associations between green space and influencing factors.

Myopia, the most common refractive error leading to short-sightedness or nearsightedness [1,2], affects daily life and increases the risk of blindness [3,4]. This serious public health issue is likely to affect approximately half of the world's population by 2050 [5]. Therefore, knowledge of the factors affecting myopia is essential. The genetic and nongenetic factors underlying myopia have been examined [6,7], and nongenetic factors (multiple types) may be more important [8,9]. Research has revealed that urbanization [10,11], outdoor exercise [12-14], parental myopia [15,16], near-work (sustaining a close gaze for reading and writing, using computers, or using small electronic devices) time, body posture [17,18], sleep [19,20], eye care, and diet [21] all impact myopia. These influences vary with demographics [9,22,23] and geographical location [1]. Green space is associated with these factors (Figure 1). Urbanization is considered a risk factor for myopia, possibly due to less green space in urban areas than in suburban or rural areas. Outdoor exercise is a protective factor. More green space promotes more outdoor exercise [24,25] and may reduce near-work time [14]. Moreover, people with higher levels of green space have a lower prevalence of insufficient sleep [26,27]. Hence, greenness may contribute to protection against myopia.

The school is the principal space for school-aged children and adolescents. Previous studies have shown that the greenness level of a campus and the surrounding environment significantly impact students' physical and mental health [28-30]. Recently, researchers have investigated the relationship between school green space and students’ myopia [31-33]. Studies focusing on the outdoor surrounding greenness within or around school boundaries have found a negative association between green space exposure and myopia among the school-aged population [34]. However, the evidence for this effect on junior and senior high school students is weak (eg, insufficient study quantity, lacking consideration of trees’ distribution in green space). Therefore, there are still gaps in the controlling strategies for high school–aged students’ myopia with regard to green space as a protective factor, and more research is warranted.

Our study focused on high school–aged adolescents in Beijing, a population with an alarming prevalence of myopia (once reaching 98.27% in a cross-sectional investigation of senior grade 3 students) [35-37]. We hypothesized that the greenness within or around the school can protect against myopia in the junior and senior high school–aged population. We aimed to determine (1) whether greenness significantly varies between myopia and nonmyopia groups, (2) how greenness acts on individual myopia risk and school myopia prevalence after adjusting for other known influential factors, and (3) whether the greenness protective effect is similar among demographic subgroups. A clarification of these points could elucidate how green space affects myopia in the high school–aged population and potentially contribute to targeted myopia prevention and intervention measures.

## Methods

### Study Population

The study sample was taken from the monitoring project for common diseases (eg, myopia, obesity) in students and health influence factors conducted by the Beijing Municipal Health Commission in 2021. Various schools involved in this monitoring project were randomly selected from all districts of Beijing. Our study only focused on high school adolescents (junior and senior high schools). We obtained myopia and questionnaire data from 51 high schools and 13,380 students. Among the 51 schools, 28 (55%) schools were junior high schools, 20 (39%) were senior high schools, and 3 (6%) contained both junior and senior students. Since these 3 school campuses were shared by junior and senior students, there were 48 school plots in total (Figure S1 in Multimedia Appendix 1).

### Ethical Considerations

The study was approved by the Ethical Committee of the Peking University Third Hospital (IRB00006761-M2021281) and conducted according to the guidelines of the Declaration of Helsinki.

### Myopia Determination

The students included in this study underwent an ophthalmological examination at the school infirmary. The examinations were conducted in accordance with the standards of myopia screening for children and adolescents issued by the Beijing Center for Disease Prevention and Control. The examinations included far-vision examination and refractive examination and were performed by an ophthalmologist having a Chinese national medical practitioner certificate in ophthalmology. Refraction was detected using a desktop noncycloplegic autorefractometer, and all refractometers were accredited to the ISO 10342:2010 standard for ophthalmic instruments and eye refractometers. Adolescent myopia was defined as a spherical equivalent of ≤–1.00 diopters in the worse eye. The worse eye was defined as the eye with the greatest absolute value of the spherical equivalent of the refractive error.

### School Green Space

We used the normalized difference vegetation index (NDVI) to measure the schools’ green space. The NDVI value ranges from –1 to 1; the higher the NDVI, the higher the level of school green space. GF-2 satellite imagery (multispectral with 3.2 m resolution) was used in our study to calculate the NDVI values. In total, 25 single GF-2 cloud-free images (entirely covering 48 school plots and within 2 years around the myopia data collection time) were selected (data searched from the China Center for Resources Satellite Data and Application [38]). Through radiometric calibration, atmospheric correction and orthorectified (PRC parameters), these images were used to determine the NDVI by brand calculation, according to the following formula: NDVI = (NIR –RED)/(NIR + RED), where RED is the red band and NIR is the near-infrared band. Next, we extracted each plot’s average NDVI of buffers (ie, 500 and 1000 m) around the school (Figure S1 in Multimedia Appendix 1). In addition, we analyzed the positions of trees relative to school (teaching) buildings by distinguishing the distribution of trees separated from the buildings with a small space from that with a large space.

### Individual Factors

Demographic data were obtained, including birth data (used to convert the sample’s age by the current month and year plus the birth month and year), gender (male and female categories), grade (representing exposure time to the school environment: senior/junior grade 1, indicating 1 year of exposure time; senior/junior grades 2 and 3, representing 2 and 3 years of exposure time, respectively), nationality (Han nationality and other categories), place of origin (urban, ie, 6 core urban districts: Dongchen, Xicheng, Haidian, Shijingshan, Fengtai, and Chaoyang [39]; suburban, ie, other 10 districts). In addition, students’ heights and weights were collected to calculate their BMI according to the “Screening for Overweight and Obesity Among School-Age Children and Adolescents” protocol (WS/*t* 586—2018).

Information about students’ outdoor exercise, including outdoor time on general days (Q1), outdoor time during holidays (Q2), preference for outdoor places at school (Q3), outdoor exercise intensity (Q4), and the number of physical education (PE) classes (Q5); near-work and body gestures, including posture-related distance from the table (Q6) and distance from the screen (Q7), adjusted chair height (Q8), parental control (Q9), and use of electronic devices (Q10); eye care, including the number of daily eye exercises at school (Q11) and the eye rest interval (Q12); dietary and sleep factors, describing sugar (Q13), fresh fruit (Q14), and fresh vegetables (Q15) consumed in the past 7 days; and the average daily sleeping hours (Q16) were acquired from the questionnaire (for details of questions Q1-Q16, see Multimedia Appendix 2).

### Statistical Analysis

We analyzed differences between myopia and nonmyopia groups at the single-factor level. Categorical variables (ie, gender, nationality, place of origin, exposure time, position of trees, outdoor exercise factors [Q1-Q3], near-work and body gesture factors [Q6-Q10], eye-care factors [Q11-Q12], and dietary factors [Q13-Q15]) were analyzed using chi-square tests to determine statistical significance, with a 2-sided *P* value of <.05 as the threshold. Numeric variables (ie, NDVI values, outdoor exercise and sleep factors (Q4, Q5, Q16), age, and BMI) were analyzed using the median Wilcoxon test (*P*<.05 was considered significant).

Generalized linear mixed models with a binomial error structure were used to test the effects of the NDVI on personal myopia risk. The NDVI and all adjusted factors (ie, demographic factors, outdoor exercise, near-work and body gestures, eye care, diet, sleep, exposure time, and the position of trees) were considered fixed effects. To control for the influence of school variation on adolescents at the personal level, we set “school” as a random factor. Moreover, to assess the influence of different NDVI ranges and their adjusted effects, we developed 6 models. Models 1a, 2a, and 3a represented the regressions of the NDVI (within the school, 500 m buffer around the school, 1000 m buffer around the school, respectively) to myopia. Based on this, adjusted factors were added to Models 1b (ie, Model 1a with the addition of adjusted factors), 2b (ie, Model 2a with the addition of adjusted factors), and 3b (ie, Model 3a with the addition of adjusted factors). For the random and fixed effects for the adjusted NDVI of Models 1b, 2b, and 3b, see Figures S1 and S2 in Multimedia Appendix 3. We used the tolerance and variance inflation factor (VIF) to test collinearity among variables, with tolerance>0.1 and VIF<5 considered as no collinearity. We used the Durbin-Watson (DW) value to test the independence of residuals (with a DW value not close to 0 or 4 meaning that the variables were independent). To analyze the distinct performance of these effects in subgroups, we performed the same generalized linear mixed models under different subgroups divided by school type, gender, nationality, place of origin, and grade (converted to exposure time).

When considering the effects of the NDVI on myopia prevalence at the school level, we first calculated each school’s myopia prevalence (myopia students/total students in that school). Second, 6 generalized linear models with a quasibinomial error structure (the quasibinomial was used to replace the binomial for existing overdispersion) were developed in the same way as the regressions earlier. Models 4a, 5a, and 6a only considered the NDVI of 3 buffer scales separately, and Models 4b, 5b, and 6b were the quasibinomial regressions (weighted with the number of students in each school) of the NDVI (within the school, 500 m buffer around the school, 1000 m buffer around the school, respectively) to the school-level myopia prevalence with adjusted factors (ie, the school’s average age, gender [proportion of female students], place of origin [urban or suburban], BMI, and position of trees).

Data analyses and visualization were performed using R version 4.1.3 (R Core Team), mainly using the sjplot [40], lme4 [41], and ggplot2 [42] packages.

## Results

### Myopia Prevalence

The 13,380 students included in this study were aged 11.9-20.9 years (mean 14.86, SD 1.71), and among them, 6803 (50.84%) were male and 6577 (49.16%) female. There were 7850 (58.67%) junior high school-aged students (n=2663, 33.92%, grade 1, n=2610, 33.25%, grade 2, and n=2577, 32.83%, grade 3) and 5530 (41.33%) seniors (n=1908, 34.50%, grade 1, n=1841, 33.29%, grade 2, and n=1781, 32.21%, grade 3). Of the 13,380 adolescents, 5245 (39.20%) were from urban areas and 8135 (60.80%) from suburban areas. In addition, 12,004 (89.72%) of these students were of Han nationality, and 1376 (10.28%) were of other nationalities. According to grade, the exposure times in school were divided into 1, 2, and 3 years, accounting for 4571 (34.16%), 4451 (33.27%), and 4358 (32.57%) of the students, respectively.

The overall myopia prevalence of the high school–aged students was 80.61% (10,785/13,380, 95% CI 79.93%-81.27%). The prevalence of myopia in seniors (4754/5530, 85.97%, 95% CI 84.03%-86.86%) was higher than that in juniors (6031/7850, 76.83%, 95% CI 75.88%-77.75%); see Table 1. With the exception of nationality (*P*=.63) and BMI (*P*=.47), the prevalence of myopia varied significantly with age, gender, and place of origin (*P*<.001); see Table 1. Adolescents with myopia were more likely to be (1) older (median age 14.78 years, IQR 13.75-16.46) compared to those without myopia (median age 14.05 years, IQR 13.01-15.60), (2) schoolgirls (myopia prevalence 5548/6577, 84.4%, 95% CI 83.45%-85.21%) compared to schoolboys (myopia prevalence 5237/6803, 77%, 95% CI 75.96%-77.96%), and (3) urban students (myopia prevalence 4406/5245, 84%, 95% CI 82.98%-84.97%) compared to suburban students (myopia prevalence 6379/8135, 78.4%, 95% CI 77.5%-79.29%).

**Table 1:** Description of myopia and nonmyopia groups.

Characteristics		Total (N=13,380)	Myopia group (n=10,785, 80.61%)	Nonmyopia group (n=2595, 19.39%)	*P* value^a^	
Age (years), median (IQR)		14.63 (13.42-16.31)	14.78 (13.75-16.46)	14.05 (13.01-15.60)	<.001	
BMI (kg/m^3^), median (IQR)		21.5 (18.9-25.3)	21.4 (18.9-25.2)	21.6 (18.8-25.7)	.47	
**Gender, n (%)**						<.001
	Male	6803 (50.84)	5237 (48.56)	1566 (60.35)	N/A^b^	
	Female	6577 (49.16)	5548 (51.44)	1029 (39.65)	N/A	
**Nationality,** **n (%)**						.63
	Han nationality	12,004 (89.72)	9683 (89.78)	2321 (89.44)	N/A	
	Other nationalities	1376 (10.28)	1102 (10.22)	274 (10.56)	N/A	
**School type,** **n (%)**						<.001
	Junior	7850 (58.67)	6031 (55.92)	1819 (70.10)	N/A	
	Senior	5530 (41.33)	4754 (44.08)	776 (29.90)	N/A	
**Place of origin,** **n (%)**						<.001
	Urban area	5245 (39.20)	4406 (40.85)	839 (32.33)	N/A	
	Suburban area	8135 (60.80)	6379 (59.15)	1756 (67.67)	N/A	
**Exposure time (years),** **n (%)**						<.001
	1	4571 (34.16)	3507 (32.52)	1064 (41.00)	N/A	
	2	4451 (33.27)	3581 (33.20)	870 (33.53)	N/A	
	3	4358 (32.57)	3697 (34.28)	661 (25.47)	N/A	
**Position of trees,** **n (%)**						<.001
	From buildings with a small space	4857 (36.30)	4018 (37.26)	839 (32.33)	N/A	
	From buildings with a large space	8523 (63.70)	6767 (62.74)	1756 (67.67)	N/A	

^a^Age and the BMI were reported with *P* values in the Wilcoxon test. Other variables were reported with *P* values in the chi-square test.

^b^N/A: not applicable.

### Factors Influencing the Prevalence of Myopia

**Figure 2:**
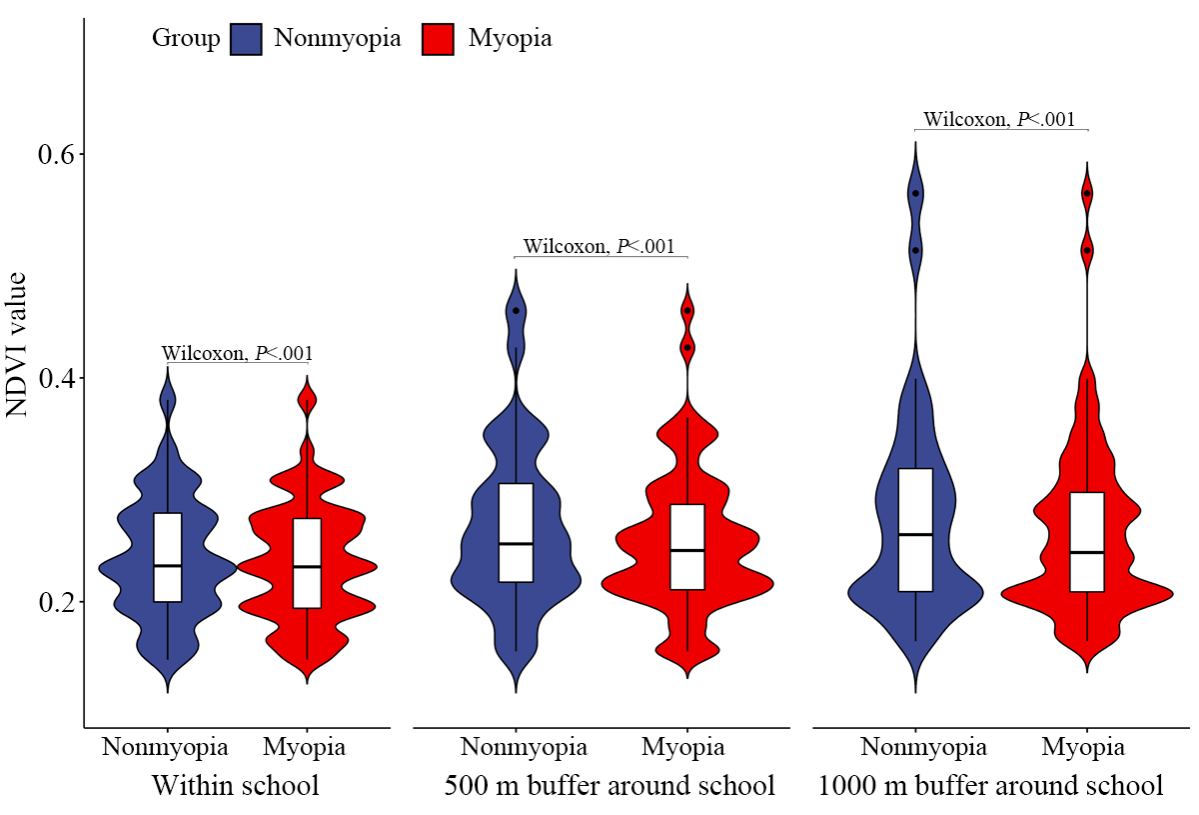
Differences in the NDVI between the myopia and nonmyopia groups. The probability density with the minimum, maximum, median, and quartiles are shown in this figure. The red color represents the myopia group, and the blue color represents the nonmyopia group. “Wilcoxon, *P*<.001” means a significant difference in the median by the Wilcoxon test. NDVI: normalized difference vegetation index.

We extracted the NDVI within each school boundary and of the 500 and 1000 m buffers (Figure S2 in Multimedia Appendix 1). Significant differences were found in school greenness between the myopia group and the nonmyopia group (*P*<.001); see Figure 2. In the myopia group, the NDVI (1) within the school (median 0.231, IQR 0.194-0.274), (2) within the 500 m buffer (median 0.279, IQR 0.247-0.318), and (3) within the 1000 m buffer (median 0.281, IQR 0.248-0.331) was lower compared to the nonmyopia group (median 0.232, IQR 0.2-0.279; median 0.285, IQR 0.253-0.335; and median 0.296, IQR 0.249-0.351, respectively).

Furthermore, trees located too close to teaching buildings were significantly associated with a higher myopia prevalence (4018/4857, 82.73%, 95% CI 81.64%-83.77%) than trees at a distance with enough space from the buildings (6767/8523, 79.4%, 95% CI 78.53%-80.25%); see Table 1. Moreover, there were significant differences concerning other factors between students with and without myopia (*P*<.001). Students without myopia were likely associated with a higher outdoor level (Q2-Q5), less near-work time with appropriate body posture (Q6-Q8, Q10), better eye care (Q11-Q12), sufficient sleep time (Q16), and high fresh-good intake (Q14); see Multimedia Appendix 2.

### Effects of Influencing Factors on Adolescent Myopia Risk

According to the fixed effects of generalized linear mixed effect models (Table 2), the NDVI of buffers around the school significantly influenced the risk of adolescent myopia without any adjusted factors. With a 0.1 increase in the NDVI of a 500 m buffer (odds ratio [OR] 0.74, 95% CI 0.61-0.89, Model 2a) and a 1000 m buffer (OR 0.79, 95% CI 0.68-0.92, Model 3a) surrounding the school, the adolescent myopia prevalence decreased by 26% and 21%, respectively. However, the decrease in myopia prevalence caused by the 500 m (OR 0.84, 95% CI 0.73-0.97, Model 2b) or the 1000 m (OR 0.88, 95% CI 0.79-0.99, Model 3b) buffer NDVI dropped by 16% and 12%, respectively, due to the adjustment. In addition, neither the NDVI (OR 0.84, 95% CI 0.66-1.06, Model 1a) nor the adjusted NDVI (OR 0.88, 95% CI 0.75-1.04, Model 1b) within the school showed a significant association with the adolescent myopia risk.

Considering all adjusted factors (Table S1 in Multimedia Appendix 4), similar effects of age, gender, exposure time, place of origin, outdoor time during holidays (Q2), preference for outdoor place (Q3), posture-related distance from the table while doing near-work (Q6), and eye-rest interval (Q12) were found to be significantly associated with myopia when adjusting the 500 and 1000 m buffer NDVI. Of these factors, age (OR 1.13, 95% CI 1.06-1.2, Models 2b and 3b) and female gender (OR 1.52, 95% CI 1.38-1.66, Models 2b and 3b) had positive associations with myopia. A close range for reading (Q6; OR 1.22, 95% CI 1.03-1.44, Models 2b and 3b) and an eye-rest interval of 30-60 minutes compared to 15 minutes or less (Q12; OR 1.21, 95% CI 1.05-1.39, Models 2b and 3b) were risk factors for myopia. The outdoor time on holidays was negatively associated with the personal risk of myopia, and persisting in maintaining the outdoor time on holidays reduced the personal risk of myopia by 33% (OR 0.67, 95% CI 0.55-0.82, Models 2b and 3b) compared to hardly doing so. Spending break time outdoors decreased the personal risk of myopia by 10% (OR 0.90, 95% CI 0.8-0.99, Models 2b and 3b) compared to spending break time in the teaching building. In addition, suburbanization was found to lower the personal risk of myopia by approximately 20% when adjusted for NDVI effects (OR 0.81, 95% CI 0.68-0.96 for Model 2b and OR 0.8, 95% CI 0.67-0.95 for Model 3b). However, a 3-year exposure time caused a higher myopia risk than a 1-year exposure time when considering the 500 or the 1000 m buffer NDVI (OR 1.38, 95% CI 1.18-1.63, Model 2b and 3b), but no such significant difference was seen between the 2- and 1-year exposures (OR 1.12, 95% CI 0.99-1.26 for Model 2b and OR 1.12, 95% CI 0.99-1.27 for Model 3b).

**Table 2:** Summary of the NDVI^a^ effect at the personal level and the school level.

			Crude^b^					Adjusted^c^		
			OR^d^ (95% CI)^e^		*P* value		OR (95% CI)^e^			*P* value
**Personal myopia risk**										
	Within the school	0.84 (0.66-1.06)		.14		0.88 (0.75-1.04)			.15	
	500 m buffer around the school	0.74 (0.61-0.89)		.001		0.84 (0.73-0.97)			.02	
	1000 m buffer around the school	0.79 (0.68-0.92)		.002		0.88 (0.79-0.99)			.04	
**School myopia prevalence**										
	Within the school	0.85 (0.67-1.06)		.16		0.94 (0.79-1.11)			.44	
	500 m buffer around the school	0.73 (0.61-0.87)		<.001		0.85 (0.74-0.98)			.02	
	1000 m buffer around the school	0.79 (0.69-0.92)		.001		0.89 (0.80-1.01)			.05	

^a^NDVI: normalized difference vegetation index.

^b^Odds ratios (ORs) of the NDVI (within the school, 500 m buffer around the school, 1000 m buffer around the school) regressed to the personal myopia risk or the school-level myopia prevalence without multivariable adjustment.

^c^Adjusted ORs with multivariable adjustment.

^d^OR: odds ratio.

^e^OR (95% CI) of the NDVI within the school and of the 500 or 1000 m buffers around the school indicated a 0.1 change in value.

**Figure 3:**
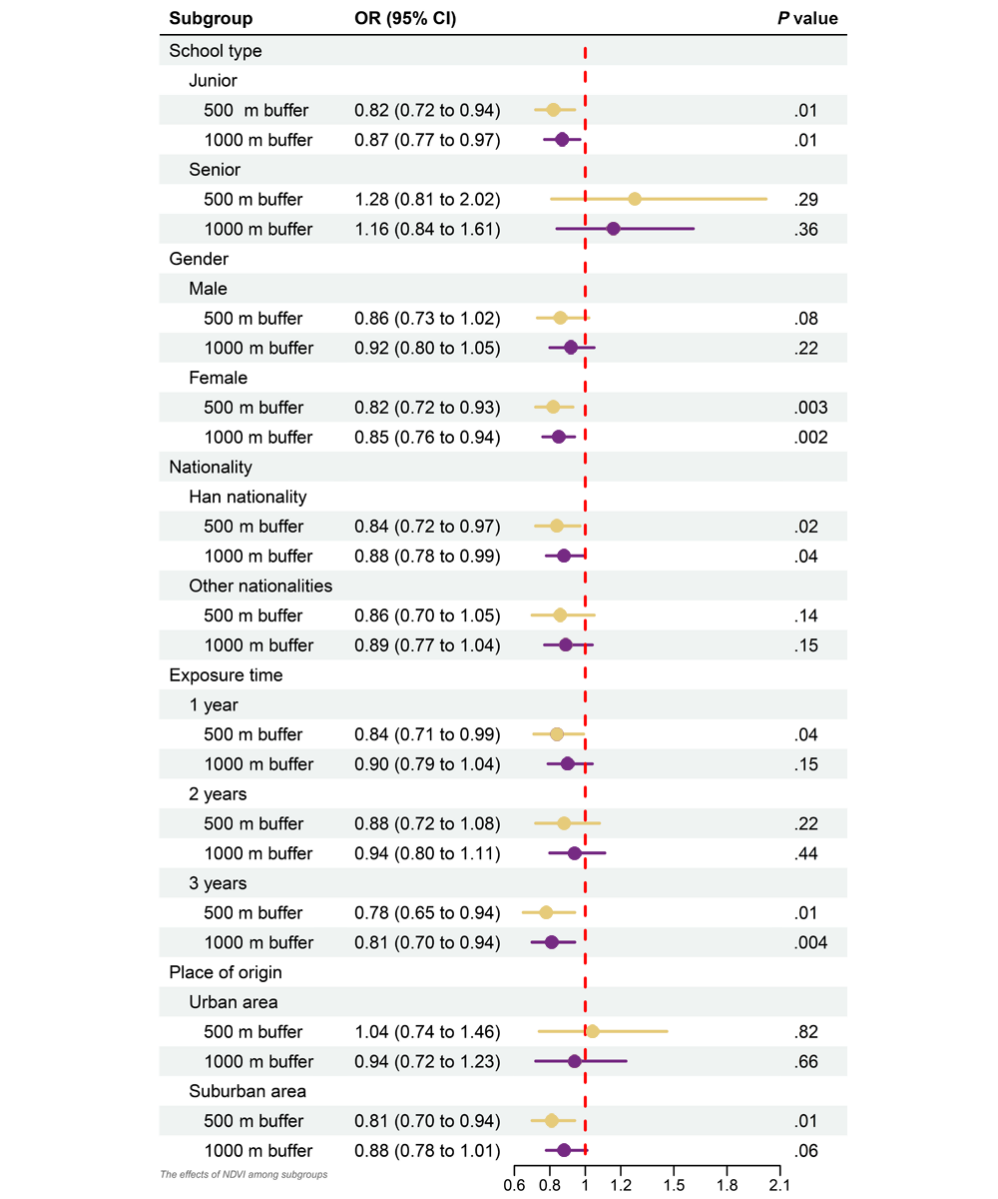
Effects of the NDVI among subgroups. The effect of the NDVI of buffers around the school were adjusted by demographic factors, outdoor exercise, near-work time, body gestures, eye care, diet, sleep factors, and the position of trees. However, this figure only shows the adjusted effects of the 500 and 1000 m buffer NDVIs surrounding the school. The lines describe the 95% CI range and the points show the OR values, while the yellow color represents the NDVI of the 500 m buffer around the school and the deep-purple color represents the NDVI of the 1000 m buffer around the school. NDVI: normalized difference vegetation index; OR: odds ratio.

Furthermore, the 500 and 1000 m buffer NDVIs performed differently in various subgroups (Figure 3). The 500 m or 1000 m buffer NDVI significantly influenced juniors (OR 0.82, 95% CI 0.72-0.94 for the 500 m buffer and OR 0.87, 95% CI 0.77-0.97 for the 1000 m buffer), schoolgirls (OR 0.82, 95% CI 0.72-0.93 for the 500 m buffer and OR 0.85, 95% CI 0.76-0.94 for the 1000 m buffer), and the the Han nationality (OR 0.84, 95% CI 0.72-0.97 for the 500 m buffer and OR 0.88, 95% CI 0.78-0.99 for the 1000 m buffer). However, only the 500 m buffer NDVI had a significant association with the suburban group (OR 0.81, 95% CI 0.7-0.94). Moreover, considering the exposure time, the NDVI of buffers around the school (OR 0.78, 95% CI 0.65-0.94 for the 500 m buffer and OR 0.81, 95% CI 0.70-0.94 for the 1000 m buffer) significantly influenced the 3-year exposure group, while only the NDVI of the 500 m buffer around the school (OR 0.84, 95% CI 0.71-0.99) significantly affect the 1-year exposure group.

### Influence of the NDVI on School-Level Myopia Prevalence

The school-level myopia prevalence was not significantly affected by the NDVI within the school (OR 0.85, 95% CI 0.67-1.06, Model 4a); see Table 2, while it was distinctly influenced by the 500 and 1000 m buffer NDVIs without any adjusted factors (Table 2). With a 0.1 increase in the 500 and 1000 m buffer NDVIs, the prevalence of myopia at the school level dropped by 27% (OR 0.73, 95% CI 0.61-0.87, Model 5a) and 21% (OR 0.79, 95% CI 0.69-0.92, Model 6a), respectively. However, after adjusting for age, gender, the BMI, place of origin, and the relative position of trees (Table S2 in Multimedia Appendix 4), only the effects of the 500 m buffer NDVI were still influential (Table 2), with its 0.1 increase significantly reducing the school-level myopia prevalence by 15% (OR 0.85, 95% CI 0.74-0.98, Model 5b). Furthermore, in this situation, the suburban (OR 0.82, 95% CI 0.65-1.02) area no longer showed a significant adjusting effect, while age still had a positive association with the school-level myopia prevalence (OR 1.22, 95% CI 1.13-1.33); see Table S2 in Multimedia Appendix 4.

## Discussion

### Principal Findings

This research shows the alarming overall prevalence of myopia in senior and junior high school students in Beijing of 80.61%(10,785/13,380, 95% CI 79.93%-81.27%). However, the protective effects of the 500 m buffer NDVI were found on both the adolescent myopia risk (OR 0.84, 95% CI 0.73-0.97) and the school-level myopia prevalence (OR 0.85, 95% CI 0.74-0.98), and it was significantly associated with schoolgirls (OR 0.82, 95% CI 0.71-0.93), juniors (OR 0.82, 95% CI 0.72-0.94), the Han nationality (OR 0.84, 95% CI 0.72-0.97), 1-year exposure (OR 0.84, 95% CI 0.71-0.99), and 3-year exposure (OR 0.78, 95% CI 0.65-0.94) myopia risk.

The prevalence of myopia in Beijing is reported to exceed the average level of other Chinese mainland regions [43,44]. However, in the past decades, a series of actions have been taken by the government to prevent myopia among school-aged children and adolescents [45]. Some studies have noted that the myopia prevalence in junior high school students and senior students dropped from 2018 to 2020 according to cohort investigations. Correspondingly, the green space area ratios of Beijing from 2018 to 2020 were 48.44, 48.46, and 48.96, respectively (Beijing Municipal Forestry and Parks Bureau [46]). Despite the lack of direct evidence to determine the associations between the decrease in the prevalence of myopia and the yearly increasing green space ratio, the benefits of a greener city for children’s and adolescents’ vision development might be meaningful because this type of relationship has been demonstrated at the national or regional level by meta-analysis [47].

Our research found important associations between the green space surrounding schools and students’ myopia, in line with our hypothesis. Thus, more attention should be paid to myopia among students in schools with less greenness around the campus environment. The importance of green space within the 500 m buffer around schools for adolescents’ myopia was emphasized in our study. Similarly, the greenness of the 500 m buffer surrounding the school has been underlined for students’ social and health benefits in other studies [48,49]. We considered that students probably tended to live close to their school to minimize time costs or benefit from opportunities offered by the school (school catchment areas probably encompass the properties nearby) [50]. Thus, the 500 m buffer might also cover the students’ outside-school environments. This situation might provide day-long exposure to greener space and reduce the risk of myopia. Moreover, a greener environment could encourage more outside activities after school, encouraging students to enhance social connections with neighbors of the same age instead of playing alone on electronic devices [51]. In addition, with more green space outside schools, students may have greener routes between school and home, and walking home along an avenue with a tree canopy could be highly inspirational [52]. Indeed, “commuting greenness” has been shown to help reduce the need for spectacles [34]. Furthermore, the marked variation in the prevalence of myopia between suburban and urban schools did not occur when the effects of the NDVI of buffers were considered. It is suggested that the greenness level around schools rather than the urbanization of the location could better determine the school prevalence of myopia. The effects of green space around schools, especially the 500 m buffer, need to be further stressed.

In addition, the effect of green space within the 500 m buffer on myopia prevalence varied in different demographic subgroups. Schoolgirls had a higher myopia prevalence and were slightly more sensitive to the 500 m buffer NDVI than schoolboys. This result showed marginalization, which is usually reported in gender variation; that is, females are marginalized with poor health outcomes but gain more than males from green space exposure [53,54]. However, some studies note that nonenvironmental factors affect myopia in schoolgirls and that gender differences are the effect of puberty hormones, which influence schoolgirls’ eye development progress and increase the tendency to develop myopia [55]. Moreover, the NDVI of the 500 m buffer around the school showed significant effects on reducing personal myopia risk both in 1- and 3-year-exposure-time subgroups, but a 3-year exposure time caused a higher myopia risk than a 1-year exposure time. Although we lack evidence about the effects of exposure time combined with the effects of green space on myopia, it could suggest that students are more likely to benefit from a school environment with adequate green surroundings. In addition, the benefit level could rapidly rise initially when they first enter, and then drop to a certain and steady level afterward. The hypothesis about this trend is also mentioned in other studies for the effects of green space on other health outcomes [56].

Our study also showed important results for other influencing factors that were similar to previous studies. Time spent outdoors has been shown to protect against myopia [14,57], and previous studies have revealed the driving mechanisms. It appears that light quality is a crucial factor in avoiding myopia development, and people with myopia have significantly lower daily light exposure [58,59]. Because the intensity of artificial light in indoor environments is much lower than that in outdoor environments [60], the shade from trees placed close to teaching buildings might make the inner classroom darker, with poor distribution of light, which may aggravate students’ refractive error problems. However, some scholars believe that prolonged exposure to a middle-level-light environment could achieve the same protection against myopia as exposure to a strong-light environment [61]. It is conceivable that the total light exposure accumulated via outdoor activities in green space with medium light intensity could effectively prevent and control myopia development, while providing a visually comfortable environment that is not harmful to the skin [62]. In addition, unlike daytime outdoor light exposure, some researchers suggest that excessive artificial light at night (ALAN) exposure results in myopia [63]. Green space has been identified as having a negative correlation with ALAN [64] and may be a potential protective factor for myopia that mitigates harmful night-light doses for children and adolescents. Furthermore, previous studies have investigated seasonal variation and myopia development [65] and have found that myopia progression is slower in the summer than in the winter. They explain this by the association between outdoor light exposure and myopia. However, these studies may have partly neglected the role of seasonal plant landscape changes with more greenery and its function in promoting more physical activity in the summer than in the winter [66]. Thus, outdoor activities in greener spaces might benefit eye development and protect against ametropia.

Importantly, this study highlighted the same beneficial factors related to good eye care, such as reduction in near-work time and better body posture, as other researchers [1]. These should be key points for myopia prevention and control in Beijing. Furthermore, some studies have summarized the control activities that should be included in government policies, school-based prevention, health sector screening and treatment, and family support for prevention [45]. In view of our findings, since adolescents lack outdoor time [67], we recommend that adequate outdoor time in green space for adolescents should be ensured by policy makers, educators, and parents. In addition, the greenness around schools (particularly within the 500 m buffer) should be improved by tree planting and canopy provision, and the government should support these measures. A higher level of greenness for schools is required because campus green space provides further health benefits (eg, improving mental well-being [28,29,68], reducing heat-related illness [69]) beyond myopia reduction. However, the vegetation distribution should be carefully considered. Creating a vision-friendly campus could include a tree cover around the playground or along inner paths, while leaving appropriate spaces between trees and building edges.

### Limitations

However, some limitations exist. First, we used cross-sectional myopia data from 2021 as many online classes were conducted during home quarantine due to the COVID-19 pandemic in 2020, which caused long periods of near-work on electronic devices and less outdoor activity time, as well as an increased prevalence of myopia [70-75]. Thus, the myopia prevalence might be overvalued with fewer environmental influences. Second, we lacked information about the green space exposure level of the samples’ previous schools (ie, the NDVI and the exposure duration of their previous schools). Therefore, the exposure time in our study might be estimated inaccurately. In addition, we did not include the myopia duration due to the lack of access to students’ previous myopia data. Third, noncycloplegic refractometry may overestimate the prevalence of myopia in teenagers with active accommodation [76]. Fourth, we did not consider other types of green space exposure, such as the accessibility and visibility of greenness.

The NDVI (2D perspective) does not precisely represent human vision greenness (3D perspective) [48], although it is an effective indicator to measure the green space level and can explain its effects on general health outcomes. Since a lack of green view is significantly related to eye fatigue, a greenness vision indicator should be considered. Future studies should address these points.

### Conclusion

To the best of our knowledge, this is 1 of the few studies to clarify the effects of school green space with buffers and other relevant factors on junior and senior high school students’ myopia prevalence. We emphasize that green space around schools is an independent protective factor for adolescents’ myopia, and we suggest the importance of the appropriate distribution of within-campus trees for myopia prevention.

## Appendix

Multimedia Appendix 1 NDVI values of study plots. NDVI: normalized difference vegetation index.

Multimedia Appendix 2 Summary of influencing factors.

Multimedia Appendix 3 Random effects and fixed effects of adjusted NDVI models. NDVI: normalized difference vegetation index.

Multimedia Appendix 4 Summary of regression resluts.

## Abbreviations

ALAN: artificial light at night
DW: Durbin-Watson
NDVI: normalized difference vegetation index
OR: odds ratio
VIF: variance inflation factor
