# Supplementary material for: Effects of Greenness on Myopia Risk and School-Level Myopia Prevalence Among High School–Aged Adolescents: Cross-sectional Study
Source: JMIR Public Health Surveill. 2023 Jan 9;9:e42694. doi: 10.2196/42694 (PMC9871879; doi:10.2196/42694)
Supplement: Multimedia Appendix 1 [file publichealth_v9i1e42694_app1.pdf]

Appendix 1

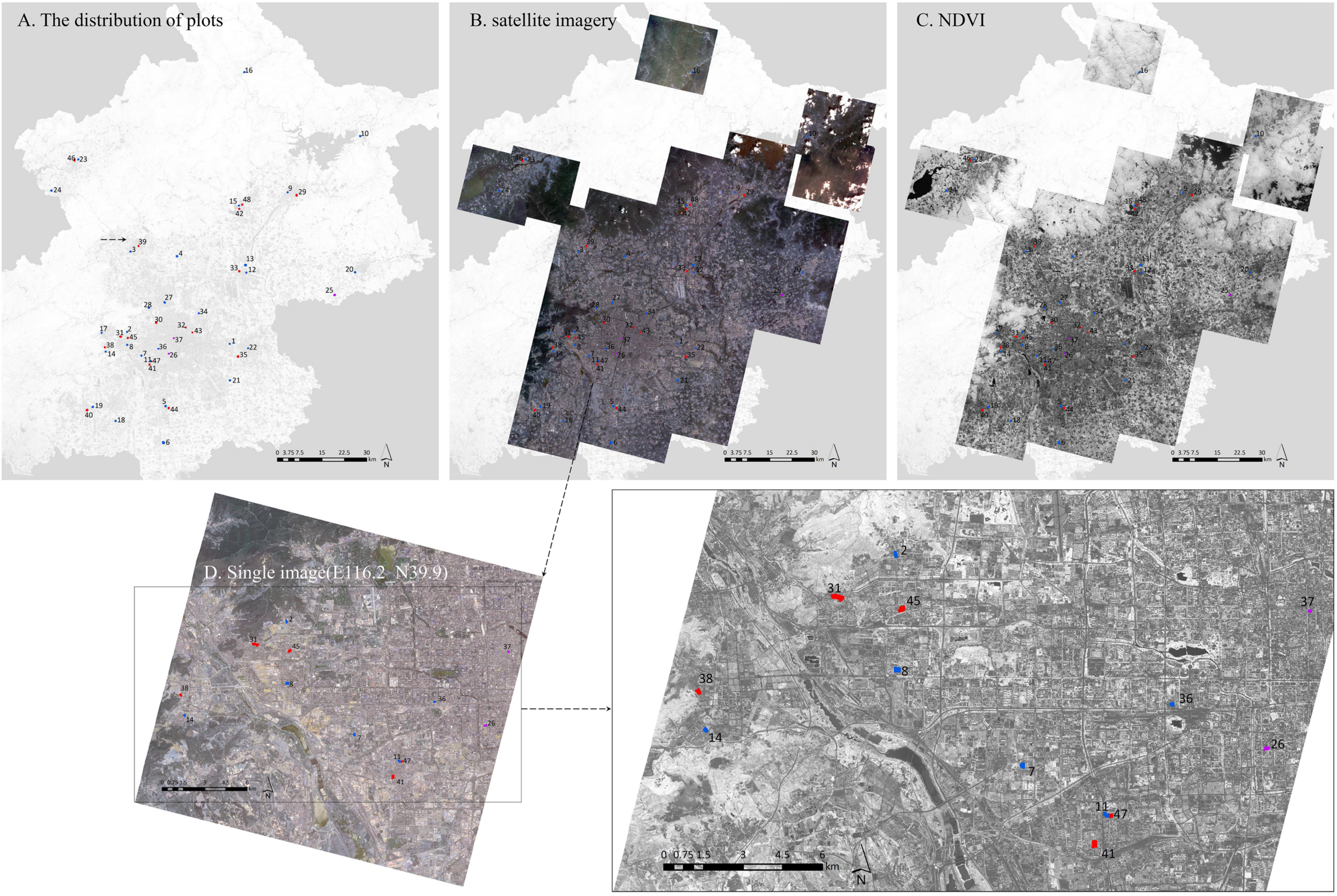

Figure S1. The distribution of the 48 study plots (A): the blue dot means the junior high schools (1-24, 27-28, 34 and 36), the red dot means the senior high schools (29-33, 35 and 38-48) and the purple dot means the school contained both junior and senior students (25, 26 and 37). A total of 25 single GF-2 image (25km *25km) covered the entire study plots (B): plots 30 and 40 were covered by two single images of Jun 2019 (E116.2_N40.0, E116.1_N39.7); six single images May of 2020 (E116.7_N39.7, E116.7_N39.9, E116.9_N40.4, E117.1_N40.2, E117.2_N40.6, E117.0_N40.0) covered the plots 9, 10, 20, 21, 22, 25, 29 and 35; plots 23 and 46 were covered by one single images of Jun 2020 (E116.0_N40.4); the plots 24 and 16 were covered by single image of Aug 2020 (E115.9_N40.4) and Jul 2021 (E116.6_N40.8), respectively; while the rest plots were covered by ten single image of Jul 2019 images (E116.6_N40.2, E116.1_N39.9, E116.4_N40.3, E116.7_N40.4, E116.5 _N40.0, E116.5_N39.9, E116.4_N39.7, E116.2_N39.7, E116.3_N40.1, E116.2_N39.9). The NDVI value (C) was calculated by NDVI=(NIR-RED)/(NIR+RED) (RED was the red band and NIR was the near infrared band). Besides, the NDVI of each plot was extracted from its corresponding single image, but the mosaic image was revealed in this figure. One of the single image (E116.2_N39.9) covering a large number of study plots was revealed (D).

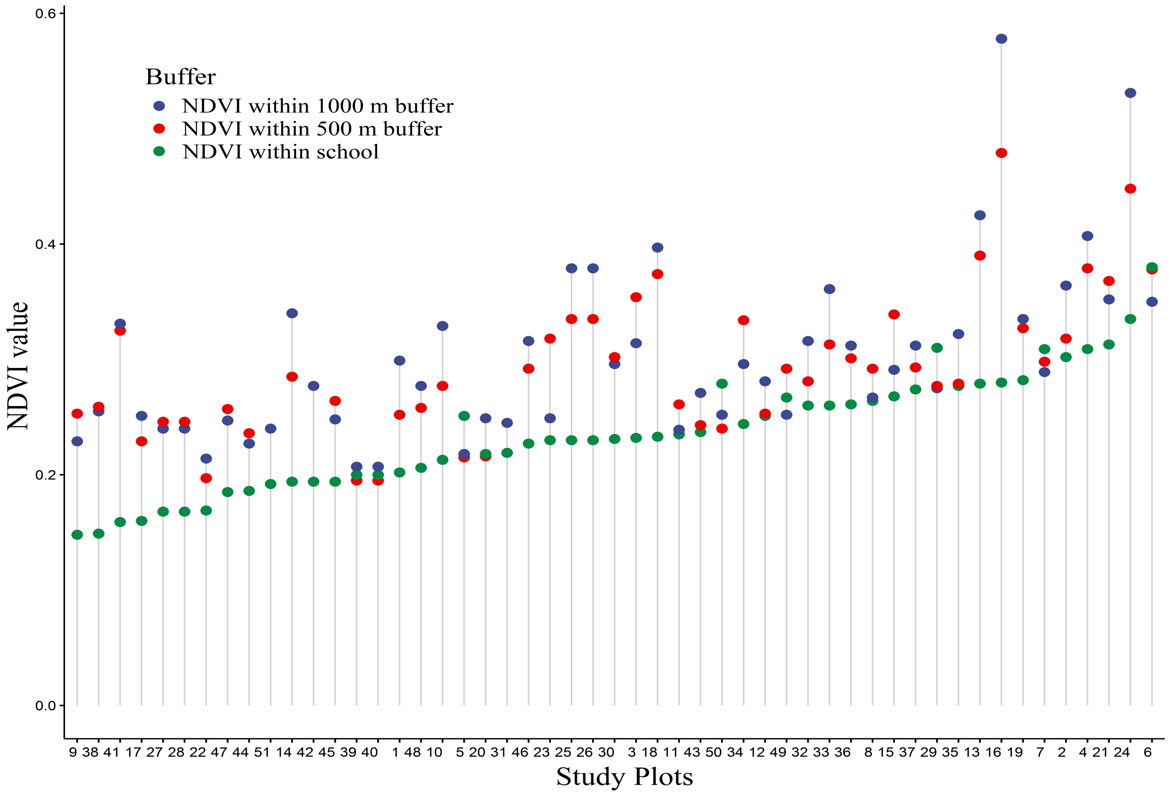

Figure S2. The NDVI value within or buffers (ie, 500 m and 1000 m) around each school plot. The green dots presented the NDVI value within each school, while the red and blue dots presented the NDVI values of 500 m and 1000 m buffers around, respectively. The average NDVI values within campus was 0.235 (SD 0.052, range 0.148-0.38), and the average NDVI values of 500 m, 1000 m buffer surrounding school were 0.289 (SD 0.059, range 0.195-0.479) and 0.3 (SD 0.073, range 0.207-0.578) respectively.
