# Supplementary material for: Effects of Greenness on Myopia Risk and School-Level Myopia Prevalence Among High School–Aged Adolescents: Cross-sectional Study
Source: JMIR Public Health Surveill. 2023 Jan 9;9:e42694. doi: 10.2196/42694 (PMC9871879; doi:10.2196/42694)
Supplement: Multimedia Appendix 2 [file publichealth_v9i1e42694_app2.pdf]

Appendix 2
Summary of influence factors

Influence factors [a]	Sample N=13380	Myopia n=10785	Nonmyopia n=2595	*P* value [b]
Q1_How long have you do outdoor exercises during the daytime in the past 7 days? , n(%)				.07
Less than 1 hour,	2513	2045 (81.4)	468 (18.6)	
1-2 hours	5867	4768 (81.3)	1099 (18.7)	
2-3 hours	2344	1865 (79.6)	479 (20.4)	
3 hours more	2656	2107 (79.3)	549 (20.7)	
Q2_Can you do exercise for more than 60 minutes on weekends and holidays. , n(%)				< .001
Absolutely can do that	2346	1740 (74.2)	606 (25.8)	
Most times can do that	3235	2571 (79.5)	664 (20.5)	
Half times can do that	2854	2325 (81.5)	529 (18.5)	
Few times can do that	3500	2909 (83.1)	591 (16.9)	
Hard to do that	1445	1240 (85.8)	205 (14.2)	
Q3_Where do you usually prefer to stay during a break at school? , n(%)				< .001
In the teaching building	10554	8608 (81.6)	1946 (18.4)	
outdoors	2826	2177 (77)	649 (23)	
Q4_How many days can you do at least 60 minutes of medium to high intensity exercise in the past 7 days (cumulative)? , median (IQR)	5(2-6)	4(2-6)	5(3-7)	< .001
Q5_How many physical education (PE) classes have you had in the past 7 days? , median (IQR)	3(3-4)	3(3-4)	4(3-4)	< .001
Q6_Do you keep more than the width of a fist between your chest and the table when reading or writing? , n(%)				< .001
never	1277	996 (78)	281 (22)	
sometimes	5178	4324 (83.5)	854 (16.5)	
often	3830	3076 (80.3)	754 (19.7)	

Influence factors [a]	Sample N=13380	Myopia n=10785	Nonmyopia n=2595	*P* value [b]
always	3095	2389 (77.2)	706 (22.8)	
Q7_Is the distance between your eyes and the screen more than 66cm (approximately the length of 3 spring water bottles) when using computer or other electronic devices? , n(%)				< .001
Never	1218	924 (75.9)	294 (24.1)	
Sometimes	3505	2901 (82.8)	604 (17.2)	
Often	3107	2567 (82.6)	540 (17.4)	
Always	3550	4393 (79.2)	1157 (20.8)	
Q8_Will the height of the desk and chair be adjusted according to your stature? , n(%)				< .001
never	1693	1398 (82.6)	295 (17.4)	
Once a year	1515	1240 (81.8)	275 (18.2)	
Once a semester	5133	4181 (81.5)	952 (18.5)	
Once every 2-3 months	5039	3966 (78.7)	1073 (21.3)	
Q9_Do parents limit the time you spent on the TV, computer or video games? , n(%)				.21
Yes	7185	5762 (80.2)	1423 (19.8)	
No	6195	5023 (81.1)	1172 (18.9)	
Q10_Have you used electronic devices in the past 7 days, n(%)				< .001
No	2224	1721 (77.4)	503 (22.6)	
Yes	11156	9064 (81.2)	2092 (18.8)	
Q11_How many times do you do eye exercises at school in one day? , n(%)				.004
None in school	354	289 (81.6)	65 (18.4)	
Once	980	819 (83.6)	161 (16.4)	
Twice	10557	8518 (80.7)	2039 (19.3)	
More than 3 times	1489	1159 (77.8)	330 (22.2)	
Q12_How often do you rest your eyes during near work (by looking far into the distance, closing your				< .001

Influence factors [a]	Sample N=13380	Myopia n=10785	Nonmyopia n=2595	*P* value [b]
eyes or doing outdoor activities)? , n(%)				
Within 15 minutes	1828	1385 (75.8)	443 (24.2)	
15 to 30 minutes	5447	4382 (80.4)	1065 (19.6)	
0.5-1 hour	5131	4223 (82.3)	908 (17.7)	
1-2 hour	974	795 (81.6)	179 (18.4)	
Q13_How many times have you had sugary drinks in the past 7 days (such as Coke)? , n(%)				.43
Never	3751	3005 (80.1)	746 (19.9)	
Less than once a day	8756	7085 (80.9)	1671 (19.1)	
More than once a day	873	695 (79.6)	178 (20.4)	
Q14_How many times have you had fresh fruit in the past 7 days? , n(%)				.007
Never	506	387 (76.5)	119 (23.5)	
Less than once a day	2753	2232 (81.1)	521 (18.9)	
More than once a day	6761	5502 (81.4)	1259 (18.6)	
More than twice a day	3360	2664 (79.3)	696 (20.7)	
Q15_How many times have you had fresh vegetables in the past 7 days? , n(%)				.41
Never	253	198 (78.3)	55 (21.7)	
Less than once a day	1029	817 (79.4)	212 (20.6)	
More than once a day	3764	3021 (80.3)	743 (19.7)	
More than twice a day	8334	6749 (81)	1585 (19)	
Q16_The average daily sleep time , (median, IQR)	7(6-8)	7(6-8)	8(7-8)	< .001

Notes: [a]Q1-Q5 for outdoor exercise factors; Q6-Q10 for near-work and body gesture factors; Q11-Q12 for eye care factors; Q13-Q16 for dietary and sleep factors. [b] Q4, Q5 and Q16 were reported with *P*-values in Wilcoxon test. Other variables were reported with *P*-values in chi-square test.
