# Supplementary material for: Effects of Greenness on Myopia Risk and School-Level Myopia Prevalence Among High School–Aged Adolescents: Cross-sectional Study
Source: JMIR Public Health Surveill. 2023 Jan 9;9:e42694. doi: 10.2196/42694 (PMC9871879; doi:10.2196/42694)
Supplement: Multimedia Appendix 3 [file publichealth_v9i1e42694_app3.pdf]

Appendix 3

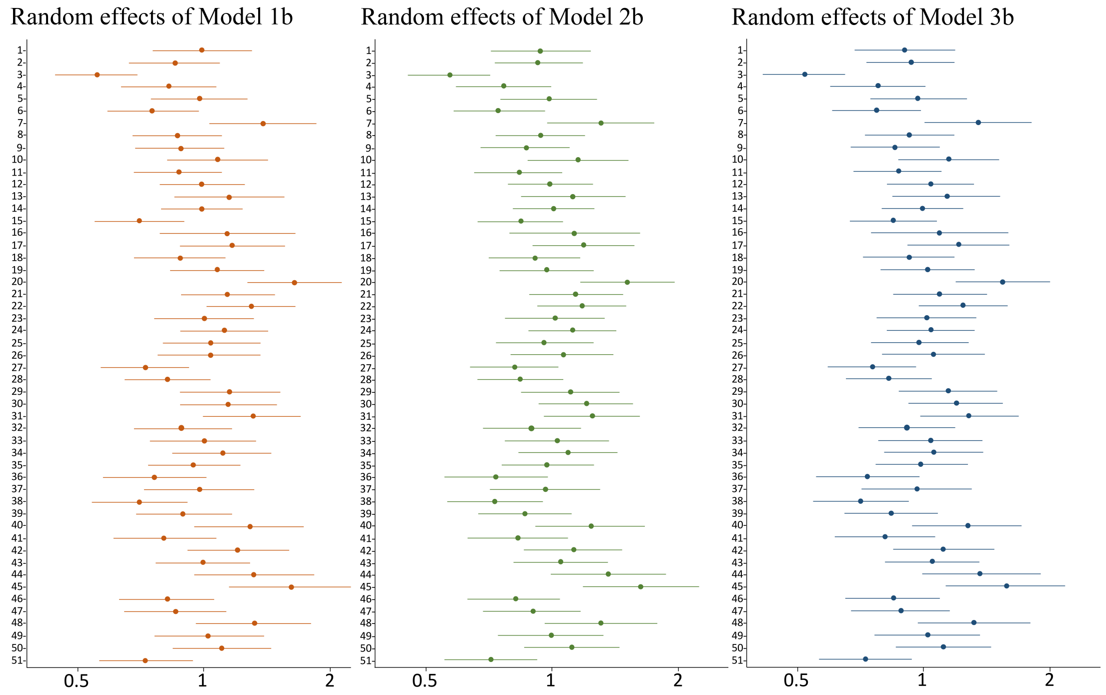

Figure S1. The random effects of generalized linear mixed effect models. The random effects were the influence of schools in generalized linear mixed effect models.

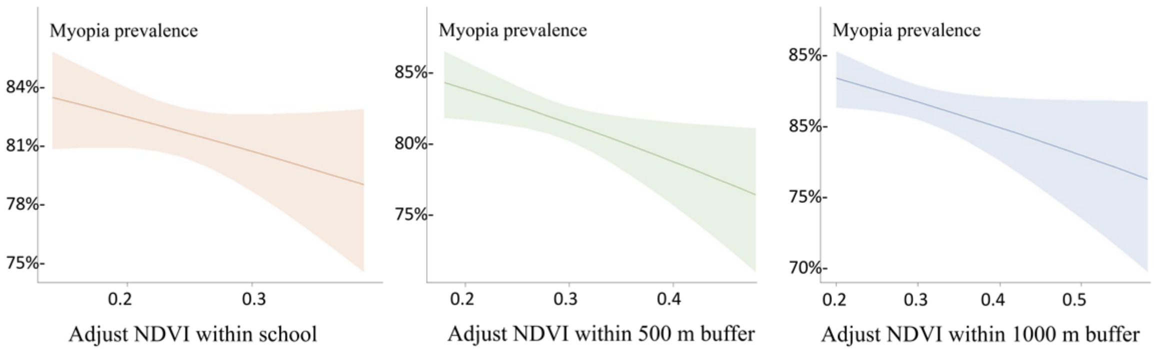

Figure S2. The fixed effects for adjusted NDVI of generalized linear mixed effect models. The color organ, green and blue represent Modle1b, Modle2b and Model3b, respectively.
