# Supplementary material for: Effects of Greenness on Myopia Risk and School-Level Myopia Prevalence Among High School–Aged Adolescents: Cross-sectional Study
Source: JMIR Public Health Surveill. 2023 Jan 9;9:e42694. doi: 10.2196/42694 (PMC9871879; doi:10.2196/42694)
Supplement: Multimedia Appendix 4 [file publichealth_v9i1e42694_app4.pdf]

Appendix 4

Table S1. Summary of the generalized linear mixed effect models [a-c]

Variables	Model 1a		Model 2a		Model 3a	
	OR (95% CI)	*P* value	OR (95% CI)	*P* value	OR (95% CI)	*P* value
**NDVI**						
Within school	0.84 (0.66,1.06)	0.143	--		--	
500 m buffer around school	--		0.74 (0.61,0.89)	0.001	--	
1000 m buffer around school	--		--		0.79 (0.68,0.92)	0.002
	Model 1b		Model 2b		Model 3b	
**Adjusted NDVI**						
Within school	0.88 (0.75,1.04)	0.146	--		--	
500 m buffer around school	--		0.84 (0.73,0.97)	0.017	--	
1000 m buffer around school	--		--		0.88 (0.79,0.99)	0.037
**Age (in years)**	1.12 (1.06,1.19)	<.001	1.13 (1.06,1.20)	<.001	1.13 (1.06,1.20)	<.001
**Gender**						
Male	Ref.		Ref.		Ref.	
Female	1.52 (1.38,1.66)	<.001	1.52 (1.38,1.66)	<.001	1.52 (1.38,1.66)	<.001
**Place of origin**						
Urban	Ref.		Ref.		Ref.	
Suburban	0.76 (0.64,0.91)	0.003	0.81 (0.68,0.96)	0.015	0.80 (0.67,0.95)	0.012
**Exposure time**						
1 year	Ref.		Ref.		Ref.	
2 years	1.13 (0.99,1.27)	0.053	1.12 (0.99,1.26)	0.059	1.12 (0.99,1.27)	0.059
3 years	1.39 (1.18,1.64)	<.001	1.38 (1.18,1.63)	<.001	1.38 (1.18,1.63)	<.001
**Outdoor time during holidays(Q2)**						
Difficult to do	Ref.		Ref.		Ref.	

Can do a few times	0.83 (0.69,0.99)	0.042	0.83 (0.69,0.99)	0.043	0.83 (0.69,0.99)	0.043
Can do half the time	0.78 (0.64,0.94)	0.009	0.78 (0.64,0.94)	0.010	0.78 (0.64,0.94)	0.010
Can do most of the time	0.79 (0.65,0.95)	0.013	0.79 (0.65,0.95)	0.014	0.79 (0.65,0.95)	0.014
Absolutely can do that	0.67 (0.54,0.82)	<.001	0.67 (0.55,0.82)	<.001	0.67 (0.55,0.82)	<.001
**Preferred place during a break(Q3)**						
In the teaching building	Ref.		Ref.		Ref.	
Outdoors	0.90 (0.80,0.99)	0.048	0.90 (0.80,0.99)	0.046	0.90 (0.80,0.99)	0.048
**Near-work body distance off table(Q6)**						
Always	Ref.		Ref.		Ref.	
Often	1.22 (1.03,1.44)	0.018	1.22 (1.03,1.44)	0.018	1.22 (1.03,1.44)	0.019
Sometimes	1.05 (0.88,1.25)	0.575	1.05 (0.88,1.25)	0.576	1.05 (0.88,1.25)	0.576
Never	1.03 (0.86,1.22)	0.777	1.03 (0.86,1.23)	0.756	1.03 (0.86,1.23)	0.754
**Eye rest interval(Q12)**						
Less than 15 min	Ref.		Ref.		Ref.	
15 to 30 min	1.07 (0.94-1.23)	0.318	1.07 (0.94,1.23)	0.316	1.07 (0.94,1.23)	0.317
0.5-1 hour	1.21 (1.05,1.39)	0.008	1.21 (1.05,1.39)	0.008	1.21 (1.05,1.39)	0.008
1-2 hours	1.13 (0.92,1.39)	0.236	1.13 (0.92,1.39)	0.230	1.13 (0.92,1.39)	0.230

Notes: [a]Model 1a, Model 2a, and Model 3a represent the generalized linear mixed effect models of NDVI (within school, 500 m buffer around school, 1000 m buffer around school, respectively) regressed to myopia. Based on this, adjusted demographic factors, outdoor exercise, near-work and body gestures, eye care, diet, sleep, exposure time, and the position of trees were added to Model 1b (ie, Model 1a adding adjusted factors), Model 2b (ie, Model 2a adding adjusted factors), and Model 3b (ie, Model 3a adding adjusted factors).

[b] This table only lists the significant variables of Model1b, 2b and 3b.

[c] The OR (95% CI) of NDVI within and 500 m or 1000m buffers around school indicated a 0.1 change in value.

Table S2. Influence of factors on school-level myopia prevalence [a,b]

Variables	Model 4a		Model 5a		Model 6a	
	OR (95% CI)	*P* value	OR (95% CI)	*P* value	OR (95% CI)	*P* value
**NDVI**						
Within school	0.85 (0.67,1.06)	0.163	--		--	
500 m buffer around school	--		0.73 (0.61,0.87)	<.001	--	
1000 m buffer around school	--		--		0.79 (0.69,0.92)	0.001
	Model 4b		Model 5b		Model 6b	
**Adjusted NDVI**						
Within school	0.94 (0.79,1.11)	0.436	--		--	
500 m buffer around school	--		0.85 (0.74,0.98)	0.024	--	
1000 m buffer around school	--		--		0.89 (0.80,1.01)	0.054
**Age (years)**	1.22 (1.13,1.33)	<.001	1.22 (1.13,1.33)	<.001	1.22 (1.13,1.33)	<.001
**Gender**						
Male	Ref.		Ref.		Ref.	
Female	0.72 (0.09,5.66)	0.758	0.81 (0.12,5.61)	0.834	0.90 (0.12,6.57)	0.914
**BMI (kg/m2)**	0.97 (0.83,1.13)	0.712	0.97 (0.83,1.11)	0.601	0.97 (0.83,1.12)	0.659
**Place of origin**						
Urban	Ref.		Ref.		Ref.	
Suburban	0.77 (0.61,0.96)	0.024	0.82 (0.65,1.02)	0.078	0.81 (0.64,1.01)	0.068
**Position of trees**						
From buildings with a small space	Ref.		Ref.		Ref.	
From buildings with large space	1.12 (0.92,1.35)	0.268	1.07 (0.89,1.28)	0.499	1.07 (0.88,1.29)	0.509

Notes: [a]Model 4a, Model 5a, and Model 6a represent the logistic regressions of NDVI (within school, 500 m buffer around school, 1000 m buffer around school, respectively) to the school-level myopia prevalence. Based on this, the adjusted factors of age, gender, BMI, place of origin and position of trees were added to Model 4b (ie, Model 4a adding adjusted factors), Model 5b (ie, Model 5a adding adjusted factors),

and Model 6b (ie, Model 6a adding adjusted factors).
[b] The OR (95%) of the NDVI within and 500 m or 1000 m buffers around school indicated a 0.1 change in value.
